# The relationship between transmission misinformation, COVID-19 stress and satisfaction with life among adults

**DOI:** 10.3389/fpsyg.2022.1003629

**Published:** 2023-02-09

**Authors:** Phuong Thi Hang Nguyen, Son Van Huynh, Nhi Ngoc Yen Nguyen, Tran Bao Le, Pha Cam Le, Gallayaporn Nantachai, Vinh-Long Tran-Chi

**Affiliations:** ^1^Faculty of Psychology and Education, The University of Danang – University of Science and Education, Da Nang, Vietnam; ^2^Faculty of Psychology, Ho Chi Minh City University of Education, Ho Chi Minh City, Vietnam; ^3^Department of Psychiatry, Faculty of Medicine, Chulalongkorn University, Bangkok, Thailand

**Keywords:** adult, COVID-19 stress, frontline health worker, satisfaction with life, transmission of misinformation

## Abstract

The perplexing evolution of the COVID-19 pandemic has had a significant effect on the spiritual lives of Vietnamese people in general, and particularly adults. The objective of this study was to ascertain the link between adult satisfaction with life and COVID-19 stress in Vietnam and investigate if COVID-19 transmission disinformation modifies the effect of COVID-19 stress on adult satisfaction with life. A total of 435 Vietnamese adults were enrolled online to finish answering, including the Satisfaction with Life Scale (SL), the COVID-19 Stress Scale (CS), and the COVID-19 Transmission Misinformation Scale (CTMS), consisting of 350 females and 85 males. Correlation, regression, and basic mediation analyses were used to dissociate the data. According to the findings of our study, there is a difference in gender in satisfaction with life. Females have a greater degree of satisfaction with life than males. Significant differences exist between relatives of direct and indirect COVID-19 transmission misinformation workers. People who had relatives who were frontline medical staff had higher COVID-19 Transmission Misinformation than others. There is a positive correlation between satisfaction with life and COVID-19 spreading disinformation, but it can have adverse effects on persons’ physical health. Additionally, COVID-19 transmission misinformation has a role in the relationship between COVID-19 stress and adult life satisfaction. Individuals are more likely to access misinformation about COVID-19 transmission, which results in enhanced life satisfaction. During the COVID-19 epidemic, adults in Vietnam should be aware of the damaging consequences of COVID-19 transmission misinformation on their stress levels. Stress may significantly influence not just one’s mental health but also other aspects of one’s life. Clinicians should be aware of COVID-19 transmission misinformation and stress, which affect psychological treatment.

## Introduction

In December 2019, a cluster of pneumonia instances that are out of the ordinary was identified in Wuhan, China. On February 11, 2020, the World Health Organization (WHO) recognized the cluster as Coronavirus disease 2019 (COVID-19) (Anand et al., 2020). The COVID-19 Pandemic has seen the rapid and widespread sharing of transmission misinformation globally, which has had detrimental effects on containment efforts and the public (Zarocostas, 2020; Anastasiades et al., 2021). Stress was recorded in the general population in China, Spain, Italy, Iran, the United States, Turkey, Nepal, and Denmark during the COVID-19 Pandemic (8.1 to 81.9%) (Xiong et al., 2020). Numerous recent studies have discovered global increases in the prevalence and severity of COVID-19-related melancholy, and stress (Asmundson et al., 2020; Xiong et al., 2020), all of which are most likely the result of barriers and adaptations made to daily life to limit viral transmission (Pfefferbaum and North, 2020). The levels of satisfaction with life have been low during the pandemic (Preetz et al., 2021). The fourth pandemic wave in Vietnam, which lasted from April 27 until now, has drastically altered society. This epidemic is considered the most severe and has resulted in the most deaths (Quan et al., 2021a). Two million seven hundred eighty-seven thousand four hundred ninety-three instances were documented from April 2021 to February 2022, with 39,423 deaths in Vietnam (World Health Organization, 2022). In December 2021, a fresh COVID-19 variant will be released. During the outbreak, Omicron was more aggressive and infectious than prior strains. Since the first incidence of Omicron VOC was reported on December 27, 2021, there have been 205 cases documented in Vietnam as of February 20, 2022 (World Health Organization, 2022). According to recent examinations by the Chinese Psychological Association and groups of Taiwanese and Vietnamese professionals, 18,000 people have been diagnosed with COVID-19 stress (Nguyen and Le, 2021). The COVID-19 pandemic and its response have tested people’s mental health, leaving them stressed and overwhelmed and ultimately contributing to destructive emotions. Social media influence people’s perceptions of the natural world. Transmission misinformation on social media can distort reality and have unanticipated repercussions. It can vary from the content of pandemic situations, COVID-19 treatments to vaccinations or antibiotics (Quan et al., 2021a). COVID-19 affects people’s satisfaction with life reported in recent studies (Than et al., 2020; Tran et al., 2020; Quan et al., 2021b). Satisfaction with life was reduced among Vietnamese university students (Duong, 2021).

### COVID-19 stress and satisfaction with life

The COVID-19 pandemic caused increased stress to the general populace, resulting in reduced satisfaction with life (Bukhari et al., 2021; Ocaña-Moral et al., 2021). During the Pandemic, physical and mental health were proven to be a strong positive predictor of life (Lee et al., 2021). However, little research has been published on the effect of the COVID-19 epidemic on people’s satisfaction with life. Countries used large-scale containment measures like “social separation” and “stay-at-home” orders to bring the COVID-19 outbreak to an end (Jacobson et al., 2020). The current focus on COVID-19 virus propagation worldwide is likely to divert public attention away from the outbreak’s psychosocial repercussions on both affected individuals and the general population. The mental health concerns resulting from this global catastrophe lead to long-term health issues, isolation, and stigma (Torales et al., 2020).

The study outcomes reveal that subjective stress and satisfaction with life are statistically significant and that there is a moderate avoidant association between them (Demir et al., 2021). There is some proof that COVID-19-associated stress is negatively connected to life satisfaction and that related dimensions such as loneliness (Groarke et al., 2020) and posttraumatic stress disorder (Karatzias et al., 2020) were elevated during the pandemic lockdown’s initial phase. The negative association between COVID-19 stress and satisfaction with life may be particularly severe for people in small and large households, especially for those living alone (Oh and Neal, 2021).

### COVID-19 stress and COVID-19 transmission misinformation

An infodemic is an overabundance of information, some of which is true and some of which is not, making it difficult for individuals to access reliable sources and credible assistance when they are in need (World Health Organization, 2020). It has a negative impact on clients’ mental health as well as encourages dangerous behavior (Luqman et al., 2017). A recent study has shown that transmission misinformation about COVID-19 is one of the stressors (Garcini et al., 2021). COVID-19 transmission misinformation, as well as that of other players, can add to stress and mental morbidity (Zandifar and Badrfam, 2020). Seeking information is unproductive and can raise stress levels since someone is exposed to new, stressful material such as disinformation and conspiracy theories (Taylor et al., 2020).

Transmission misinformation has a significant impact on mental health by inducing fear, anxiety, and stress (Ali, 2022). Because it is directly linked to a serious health concern, transmission misinformation about COVID-19 and COVID-19 itself has been deemed a substantial source of stress and anxiety (Barua et al., 2020). There is a high potential for transmission misinformation and disinformation to spread through online sources and social media often used by the user from the Internet (Basch et al., 2020; Cuan-Baltazar et al., 2020) further contributing to stress, anxiety and depression among students (Kecojevic et al., 2020).

### Satisfaction with life and COVID-19 transmission misinformation

The COVID-19 Pandemic created excellent conditions for disinformation about the virus to flourish: high fear, low trust, and low confidence (Ahmed et al., 2020). This disinformation regarding COVID-19 manifests itself in various ways, including conspiracy theories implying that the virus was created in a laboratory for use as a biological (Barua et al., 2020). Incorrect information about COVID-19 can be dangerous because it can redirect people’s attention away from appropriate activities that would help safeguard their health and the health of others, leading them to take acts that transmit the disease or engage in other problematic behaviors (Anastasiades et al., 2021).

When news is skewed and deceptive, the negative impacts of COVID-19 media coverage on individual and population health and well-being may be amplified (Su et al., 2021). False information about COVID-19 has not only negatively impacted mental health, but it has also induced paranoia and anxiety on a global scale and in real-time (National Literacy Trust, 2018). For children and young people, false news is a severe problem, affecting their well-being and the trust in media and democracy itself, according to the United Kingdom’s Commission on Fake News and Critical Literacy in Schools (Su et al., 2021). Transmission misinformation can exacerbate our mental health concerns and amplify symptoms, making it more difficult for individuals to heal or cope better. They can also result in new mental health concerns due to the incapacity to manage both. Transmission misinformation and disinformation impede some people from carrying on with their usual lives (Kalidoss and Banerjee, 2022). When people are given incorrect COVID-19 transmission misinformation, it harms their overall well-being (Rosenberg et al., 2020; Elbarazi et al., 2022).

Through each outbreak, the COVID-19 Pandemic has had tremendous consequences and caused enormous damage to the mental health of Vietnamese people. COVID-19 cases and deaths are routinely recorded. Furthermore, the transmission of misinformation regarding COVID-19 raises people’s stress levels and satisfaction with life levels, affecting information about the Pandemic. Restrictions in isolation policies, vaccines, and so on, in particular, individuals who cannot access social support (medical, food, spiritual, ...) when they need it, also affect psychological disorders such as anxiety, stress, substance abuse, ...are conducive to the quick spread of transmission misinformation. To the study team’s knowledge, there are few studies on COVID-19 stress and transmission of misinformation in Vietnam in the setting of the raging COVID-19 Pandemic to determine if, in this context, COVID-19 stress, and misinformation are prevalent. Does COVID-19 transmission misinformation impact stress in Vietnam? And if true, how does this affect the life satisfaction of those affected by the Pandemic? Our study will investigate the relationship between COVID-19 stress, the transmission of misinformation, and satisfaction with life. Across-sectional study was used to investigate the relationship between COVID-19 stress, COVID-19 transmission misinformation, and life satisfaction.

*Hypothesis 1*: COVID-19 stress would be negatively correlated with satisfaction with life.

*Hypothesis 2*: COVID-19 transmission misinformation would be related positively to COVID-19 stress.

*Hypothesis 3*: Satisfaction with life would be positively connected with COVID-19 transmission misinformation.

*Hypothesis 4*: COVID-19 stress (a) and COVID-19 transmission misinformation (b) would predict satisfaction with life.

*Hypothesis 5*: COVID-19 transmission misinformation may mediate between COVID-19 stress and satisfaction with life.

## Materials and methods

### Participants

In total, 435 Vietnamese adults volunteered to take part in the research. The sample consists of 350 (80.5%) females and 85 (19.5%) males, with most of them having received two injections (*n* = 362; 83.22%), followed by booster shots have been given (*n* = 60; 13.79%), one injection (*n* = 9; 2.06), and not yet injected (*n* = 4; 0.91%). A total of 95.86% were not infected with COVID-19, and 4.14% were infected. Most (91.03%) used a medical mask, 5.06% used a cloth mask, and 3.92% used an N95 mask. Table 1 shows the whole participant distribution.

**Table 1:** Sample socio-demographic characteristics.

Variables	Frequency (%)
Gender	
Female	350 (80.5)
Male	85 (19.5)
*Do you have a relative who is a frontline health worker performing direct and indirect work?*	
Yes	108 (24.8)
No	327 (75.2)
*Do you have chronic diseases?*	
Yes	60 (13.79)
No	375 (86.21)
*You have been vaccinated against COVID-19*	
Not injected yet	4 (0.92)
Have had one injection	9 (2.07)
Have had two injections	362 (83.22)
Booster shots have been given	60 (13.79)
Type of vaccine technology you have/will get one injection	
Vector technology	282 (64.83)
mRNA technology	52 (11.95)
Inactivation technology	101 (23.22)
The type of vaccine technology you have/will get the second dose of	
Vector technology	266 (61.15)
mRNA technology	68 (15.63)
Inactivation technology	101 (23.22)
The type of vaccine technology you have/will get a third dose of	
Vector technology	268 (61.61)
mRNA technology	95 (21.84)
Inactivation technology	72 (16.55)
Infected_with_covid_19	
Yes	18 (4.14)
No	417 (95.86)
Type_of_mask	
Medical mask	396 (91.03)
Cloth mask	22 (5.06)
N95 mask	17 (3.91)
Communicate_at_close_range	
Yes	160 (38,78)
No	275 (63.22)

### Instrument and procedures

#### Instrument

##### COVID-19 stress scale

Taylor et al. (2020) developed the COVID-19 Stress Scale (CS) to investigate the psychological responses of individuals to the COVID-19 epidemic. The original CS contained 36 items that assessed five variables: (i) COVID-19 danger and contamination; (ii) COVID-19 socio-economics consequences; (iii) COVID-19 xenophobia; (iv) COVID-19 traumatic stress; (v) COVID-19 compulsive checking. CS was determined by summing all item scores. In study of Taylor et al. (2020) the Cronbach’s for each factor was 0.91, 0.91, 0.93, 0.93, and 0.89, respectively.

The current study used a 24-item questionnaire to assess four CSS-related variables: (i) COVID-19 danger; (ii) COVID-19 socio-economics consequences; (iii) COVID-19 traumatic stress and (iv) COVID-19 contamination. On a five-point scale, each item was ranked on a one-to-five Likert scale (1 = “never,” 2 = “rarely,” 3 = “sometimes,” 4 = “often,” 5 = “almost always”).

Cronbach’s α was 0.967 for the study’s overall scale. The coefficient alpha for COVID-19 danger; COVID-19 socio-economics consequences; COVID-19 traumatic stress and COVID-19 contamination was 0.928, 0.953, 0.954 and 0.952, respectively.

The current study, the CFA demonstrated that the measurement was adequate, CMIN/df = 2.502; GFI = 0.859; CFI = 0.969; TLI = 0.964; RMSEA = 0.059 and PCLOSE = 0.008 (Hair et al., 2010).

##### COVID-19 transmission misinformation scale

The COVID-19 Transmission Misinformation Scale (CTMS) was established by Bok et al. (2021). There are 12 items on the scale, with five levels: 1 = “strongly disagree,” 2 = “highly disagree,”3 = “hesitate,” 4 = “agree,” 5 = “firmly agree.”

Cronbach’s α was 0.897 for the total scale in this study. In the present study, the CFA demonstrated that the measurement was adequate, CMIN/df = 2.996, GFI = 0.945, CFI = 0.957, TLI = 0.943, RMSEA = 0.068, PCLOSE = 0.010 (Hair et al., 2010).

##### Satisfaction with life scale

Diener et al. (1985) developed the Satisfaction with Life measure, and Köker (1991) and Şimşek, 2011 tested the Turkish version for validity and reliability. The scale has five components and one dimension. The measurements were graded on a 7-point Likert scale, with 1 = strongly disagreed and 7 = strongly agree. Dymecka et al. (2021) reported Cronbach’s α = 0.87.

In this investigation, the entire scale’s Cronbach’s was 0.888. In the present study, the CFA demonstrated that the measurement was adequate, CMIN/df = 0.776, GFI = 0.997, CFI = 1, RMSEA = 0, PCLOSE = 0.873 (Hair et al., 2010).

#### Procedures

Our research gathered data *via* a survey which was conducted *via* a Google form during the lockdown, the peak period of Vietnam’s terrible epidemic. The sample members were given online survey forms through Facebook, Zalo, or email or teachers would send online surveys to their pupils. The remainder is older users with limited Internet proficiency. We will provide them with the phone connected with the survey and coach them through its completion. Data was gathered between December 28, 2021, and January 22, 2022. Before participants completed the questionnaire, they granted informed consent and discussed the poll’s rules of anonymity and confidentiality. Participants were entirely unpaid and were free to withdraw at any moment. The survey took between 20 and 25 min to complete. Participants were urged to telephone or email communication with the study team if they requested clarification at any phase during the survey.

The current study complied with the Declaration of Helsinki’s standards for human subjects’ research and the ethical principles of the American Psychological Association (APA) regarding research involving human participants.

In this study, items from three measures, including the COVID-19 Transmission Misinformation Scale (CTMS) (Bok et al., 2021), COVID-19 Stress Scale (CS) (Taylor et al., 2020), Satisfaction with Life Scale (SL) (Abdallah, 1998) were translated forward and backwards. A Vietnamese native speaker who is fluent in English translated the English version into Vietnamese before sending the Vietnamese version to a professional translator for back-translation into English (native speaker of English and fluent in Vietnamese). Finally, the study team compared the two forms (the English-translated version and the Vietnamese back-translated version) to the initial version for content accuracy and discrepancies.

#### Data analysis

The Social Sciences Statistics Program (SPSS) version 26.0 was used to process the data. To define the characteristics of the participants, descriptive statistics, validity and reliability analysis, and correlation analysis were employed. The one-way analysis of variance (ANOVA) was conducted to determine if there were any statistically significant differences between COVID-19 Transmission Misinformation and their Satisfaction with Life scores. To test the mediation hypothesis, the bootstrapping method, and the PROCESS macro (Hayes, 2017) were utilized. We used a bootstrap procedure (Shrout and Bolger, 2002) to determine the statistical significance of the hypothesized indirect impact in this study. To implement the bootstrap method, 5,000 bootstrap samples and bias-corrected 95% confidence intervals were utilized.

## Results

### Inferential analysis

Socio-Demographic Characteristics of COVID-19 Stress, COVID-19 Transmission Misinformation, and Satisfaction with Life were subjected to a one-way ANOVA to test for a significant difference, as shown in Table 2. In the COVID-19 Transmission Misinformation and Satisfaction with Life survey, there was a large disparity between male and female. The females (*M* = 2.67, SD = 0.67) scoring higher than males (*M* = 1.79, SD = 0.56) on COVID-19 Transmission Misinformation. And females (*M* = 3.36, SD = 0.79) scoring higher than males (*M* = 3.10, SD = 1.01) on Satisfaction with Life. There was a significant difference between “Yes” and “No” responses to the question “Do you have a relative who is a frontline health worker performing direct and indirect work?” in the COVID-19 Transmission Misinformation. People who had relatives who were frontline medical staffs (*M* = 2.14, SD = 0.71) had higher COVID-19 Transmission Misinformation than others (*M* = 1.97, SD = 0.63).

**Table 2:** Descriptive statistics.

Variables	Mean ± SD	Mean ± SD	Mean ± SD
*Gender*	CS	CTMS***	SL*
Female	2.25 ± 0.82	2.67 ± 0.67	3.36 ± 0.79
Male	2.08 ± 0.82	1.79 ± 0.56	3.10 ± 1.01
*Do you have a relative who is a frontline health worker performing direct and indirect work?*	CS	CTMS *	SL
Yes	2.17 ± 0.73	2.14 ± 0.71	3.38 ± 0.80
No	2.23 ± 0.85	1.97 ± 0.63	3.28 ± 0.84
*Do you have chronic diseases?*	CS	CTMS	SL
Yes	2.27 ± 0.82	2.04 ± 0.65	3.23 ± 0.90
No	2.21 ± 0.82	2.01 ± 0.64	3.31 ± 0.83
*You have been vaccinated against COVID-19*	CS	CTMS	SL
Not injected yet	2.30 ± 0.59	2.10 ± 0.30	2.7 ± 0.70
Have had one injection	2.32 ± 0.90	1.91 ± 0.50	3.24 ± 0.68
Have had two injections	2.24 ± 0.83	2.00 ± 0.65	3.32 ± 0.83
Booster shots have been given	2.04 ± 0.75	2.08 ± 0.73	3.29 ± 0.92
*Type of vaccine technology you have/will get one injection*	CS	CTMS	SL
Vector technology	2.16 ± 0.82	1.98 ± 0.65	3.28 ± 0.85
mRNA technology	2.30 ± 0.84	2.08 ± 0.66	3.31 ± 0.88
Inactivation technology	2.23 ± 0.79	2.07 ± 0.68	3.38 ± 0.81
*The type of vaccine technology you have/will get the second dose of*	CS	CTMS	SL
Vector technology	2.17 ± 0.84	1.99 ± 0.68	3.30 ± 0.85
mRNA technology	2.25 ± 0.79	2.07 ± 0.61	3.22 ± 0.87
Inactivation technology	2.30 ± 0.78	2.03 ± 0.61	3.38 ± 0.81
The type of vaccine technology you have/will get a third dose of	CS	CTMS	SL
Vector technology	2.18 ± 0.82	1.95 ± 0.64	3.29 ± 0.85
mRNA technology	2.20 ± 0.81	2.10 ± 0.71	3.25 ± 0.88
Inactivation technology	2.38 ± 0.83	2.10 ± 0.62	3.43 ± 0.77
*Have you ever been infected with COVID-19?*	CS	CTMS	SL
Yes	2.35 ± 1.05	2.17 ± 0.93	3.41 ± 1.04
No	2.21 ± 0.81	2.00 ± 0.64	3.30 ± 0.83
*The type of mask that you usually use*	CS	CTMS	SL
Medical mask	2.23 ± 0.82	2.02 ± 0.66	3.29 ± 0.84
Cloth mask	2.05 ± 0.85	2.01 ± 0.61	3.26 ± 0.96
N95 mask	1.98 ± 0.68	1.87 ± 0.69	3.67 ± 0.56
*Do you often have to communicate at close range every day?*	CS	CTMS	SL
Yes	2.20 ± 0.84	2.00 ± 0.70	3.30 ± 0.85
No	2.22 ± 0.81	2.02 ± 0.63	3.31 ± 0.84

*** Significant at the 0.001 level (two-tailed).

* Significant at the 0.05 level (two-tailed).

CS, COVID-19 stress; SL, satisfaction with Life; CTMS, COVID-19 transmission misinformation.

Using the SPSS Pearson correlation analysis yields the following results: Table 3 details COVID-19 Stress, ignorance regarding COVID-19 Transmission Misinformation and Satisfaction with Life. The correlation between COVID-19 Stress and Satisfaction with Life is not statistically significant (*p* > 0.05). COVID-19 Positive but slight connection exists between stress and Transmission Misinformation (*r* = 0.287, *p* < 0.01). The connection between Satisfaction with Life and Transmission Misinformation is positive but slight (*r* = 0.127, *p* < 0.01). The conclusions regarding whether there is a correlation and the degree of correlation between the variables are based on the theory in David Nettleton’s study, which assigned a value between −1 and 1 to the correlation coefficient, where 0 represents no correlation, 1 represents total positive correlation, and 1 represents total negative correlation. This is characterized as follows: a correlation value of 0.70 between two variables indicates the existence of a substantial and positive association between the variables. A positive correlation indicates that if variable A rises, B will likewise increase, whereas a negative correlation indicates that if A increases, B will decrease. According to the study, more Transmission Misinformation is connected with higher levels of COVID-19 Stress and satisfaction with life (Nettleton, 2014). The first hypothesis should be rejected based on the data, but the second and third hypotheses should be accepted.

**Table 3:** Correlation Between COVID-19 stress, satisfaction with life, COVID-19 transmission misinformation.

Variable	CS	SL	CTMS
CS	–		
SL	0.009	–	
CTMS	0.287**	0.127**	–

** Correlation is significant at the 0.01 level (two-tailed).

CS, COVID-19 stress; SL, satisfaction with life; CTMS, COVID-19 transmission misinformation.

Prior to examining the direct and indirect effects of COVID-19 Stress on Life Satisfaction *via* the hypothesized mediators of COVID-19 Transmission Misinformation, a multiple regression analysis was conducted to test for multi-collinearity and homoscedasticity. The following independent variables were used in the multiple linear regression analysis: COVID-19 Stress, COVID-19 Transmission Misinformation, and the Satisfaction with Life Scale as the dependent variable. The preliminary assumption of multiple linear regression was used to examine multi-collinearity.

Pearson’s bivariate correlation was used to test for multi-collinearity for all independent variables. The independent variables were not multi-collinear since the correlation coefficients were less than 0.8 (Allison, 1999). There was no multi-collinearity between the independent variables in the multiple regression analysis because each variable had a tolerance of more than 0.2 and a variance inflation factor (VIF) value of less than 2 (Akinwande et al., 2015; Field, 2016). Additionally, Durbin-(DW) Watson’s statistic was used to determining the autocorrelation of independent variables, which was 1.77, suggesting that there were no significant correlations between the residuals (Field, 2016). As a result of satisfying the assumption, the regression analysis was undertaken.

Table 4 reveals that the corrected coefficient, Adjusted R2 was 0.024, suggesting that a one-unit change in the independent variable resulted in a change in the dependent variable, Satisfaction with Life. The regression model had a statistically significant coefficient of determination [*F*(2,260) = 4.245, *p* < 0.05, R2 = 0.032]. COVID-19 Transmission Misinformation (β = 0. 244, *p* < 0.01) was found to be a significant predictor of Life Satisfaction. Addition, COVID-19 Stress (β = −0.090, *p* > 0.05) was not a significant predictor of Satisfaction with Life. Therefore, the fourth hypothesis (a) was rejected and (b) was approved.

**Table 4:** Multiple regression results of satisfaction with life.

Model		Unstandardized coefficients		Standardized coefficients	*t*	*p*	*F*	*R* ^2^	Adjusted *R*^2^
		*B*	SE	**β**					
1	(Constant)	3.028	0.199		15.232	<0.001	4.245	0.032	0.024
	CTMS	0.244	0.085	0.183	2.869	0.004			
	CS	−0.090	0.067	−0.085	−1.333	0.184			

CS, COVID-19 stress; CTMS, COVID-19 transmission misinformation.

### Simple mediation models

**Figure 1:**
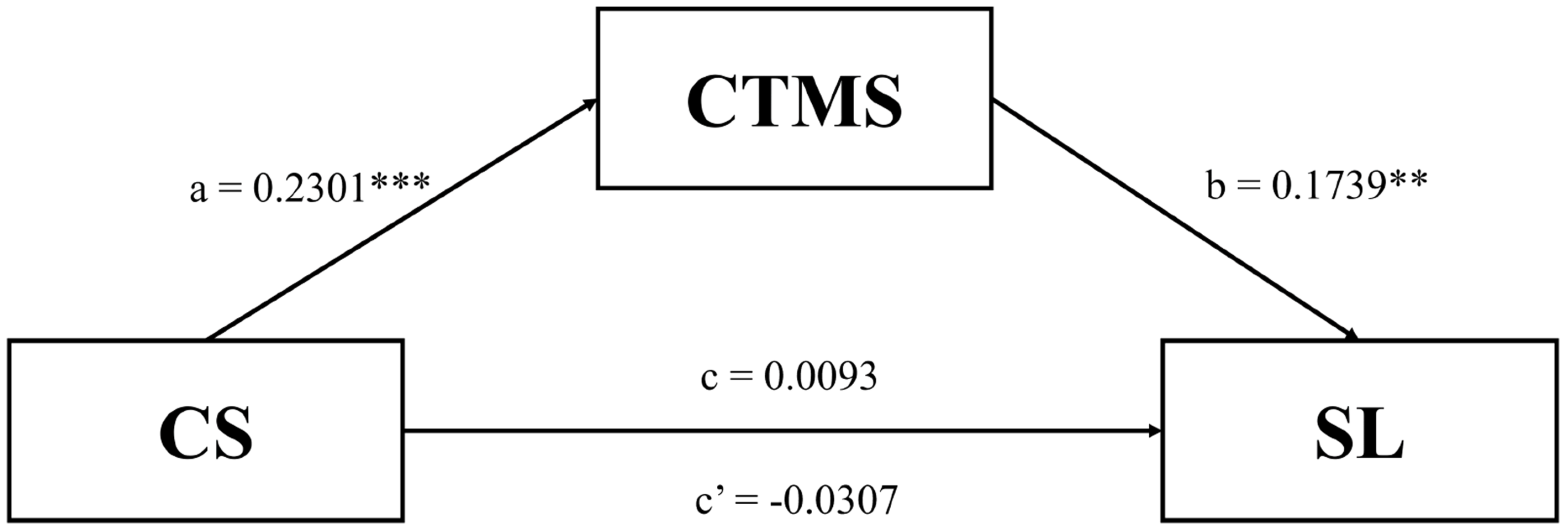
illustrates a straightforward mediation model with unstandardized coefficients. a = the cumulative impact of COVID-19 Stress on COVID-19 Misinformation Transmission. b = effect of COVID-19 Transmission Misinformation on Satisfaction with Life, when COVID-19 Stress is controlled for. c = the overall effect of COVID-19 Stress on Satisfaction with Life, unadjusted for mediators. c’ = once mediators are added in the model, the direct effect of COVID-19 Stress on Satisfaction with Life. ***p < 0.001, **p < 0.01.

We utilized a simple mediation model to investigate the indirect influence of COVID-19 Stress on Satisfaction with Life *via* COVID-19 Transmission Misinformation as addressed in Figure 1. The indirect effect is statistically significant if the 95% CI for these estimations does not contain zero (Shrout and Bolger, 2002).

Table 5 addressed that total effect (c) of COVID-19 Stress on Satisfaction with Life was not significant, *b* = 0.009, SE = 0.049, 95% CI [−0.0879–0.1066]. The direct effect (c’) of COVID-19 Stress on Satisfaction with Life was not significant, *b* = −0.0307, SE = 0.051, 95% CI [−0.1315–0.0701]. It was statistically significant that the indirect effect existed, *b* = 0.040, SE = 0.018, 95% CI [0.0081, 0.0801]. The results suggested that COVID-19 Stress on Satisfaction with Life through COVID-19 Transmission Misinformation. Therefore, the fifth hypothesis should be approved.

**Table 5:** Total, direct and indirect effects of COVID-19 stress on satisfaction with life through COVID-19 transmission misinformation.

Effects	Point estimate	SE	*t*	*p*	95% CI
Total effect	0.0093	0.049	0.188	0.851	−0.0879–0.1066
Direct effect	−0.0307	0.051	−0.599	0.549	−0.1315–0.0701
Indirect effect	0.040	0.018			0.0081–0.0801

## Discussion

The primary finding of this study was to investigate the relationship between COVID-19 stress and misinformation transmission and satisfaction with life, as well as to test whether COVID-19 misinformation transmission is a mediator of COVID-19 stress and satisfaction with life among adults in Vietnam. Our investigation yielded some noteworthy results. To begin with, females have been happier with their lives than males throughout the epidemic. Second, COVID-19 stress is not associated with low life satisfaction. Third, there was a link between COVID-19 stress and misinformation about COVID-19 transmission. Fourth, misinformation about COVID-19 transmission would be a predictor of life satisfaction. Fifth, COVID-19 transmission misinformation would act as a mediator between COVID-19 stress and life satisfaction.

The first major finding indicates that there is a considerable variation in Satisfaction with Life based on gender. This finding supports our premise that there is a gender difference in life satisfaction between males and females. This result suggests that females are happier with their lives than males. According to Joshanloo and Jovanović (2020), females were more content with their lives than males, especially in more educated populations. According to van der Laan et al. (2021), males’s life satisfaction declined significantly during the study period. The content malest of females, however, remained unchanged. Tharp et al. (2021) found no difference in life satisfaction between males and females as a result of the COVID-19 outbreak. Previous research supports our conclusion that there is a difference in life satisfaction between males and females during the COVID-19 outbreak. Several explanations have been offered as to why females report higher levels of life satisfaction than males: differences in education levels between the sexes, and differences in personality types and health states (Kruger and Engelbrecht, 2010; Joshanloo and Jovanović, 2020; Raza et al., 2020; Bermes, 2021). In addition, our findings indicate that females contribute more than males to the COVID-19 transmission misinformation. Contrary to the previous study by Bok et al. (2021) discovered that COVID-19 misinformation transmission was associated with males, younger age, college degree, and religious affiliation.

The second major finding is that COVID-19 Stress does not correlate negatively with Life Satisfaction. These results contradict our hypothesis that COVID-19 stress is negatively associated with life satisfaction. Multiple studies have produced results that support our hypothesis. Umucu et al. (2021) found that individuals with high COVID-19 stress, and low personality strengths had the lowest happiness levels. Yalçın et al. (2022) found that life satisfaction is negatively correlated with the latent profile, whose members have higher levels of COVID-19 dread, sadness, anxiety, and stress. COVID-19 stress was found to have the most negative association with life satisfaction among adults living alone or in groups (Oh and Neal, 2021). The age range 0 to 19 years comprises 40.7% of the 435 samples, whereas the age range 20 to 44 years comprises 52.9%. It is evident that the majority of the population consists of adults who reside with family or friends. This also explains why there is no negative association between COVID-19 stress and life satisfaction. Despite the fact that our findings contradict previous findings, this is an unexpected discovery nonetheless.

In addition, the data demonstrate that COVID-19 transmission misinformation is significantly and positively associated with COVID-19 stress. During this pandemic, fake news and misinformation have eroded public trust in the news, which can have negative effects on mental health (Jain, 2021). Prior research has linked misinformation on social media to a significant level of threat, which causes anxiety (Laato et al., 2020). Cuan-Baltazar et al. (2020) demonstrated that COVID-19 transmission misinformation can result in significant stress and severe mental effects. Fake news has the potential to harm society by inducing fear, anxiety, stress, and depression (Patwary et al., 2021). According to Otu et al. (2020), COVID-19 misinformation spread *via* social media increased community levels of chaos, stress, and tension. Previous research has explained how COVID-19 misinformation affects stress. According to Priego-Parra et al. (2020) the misuse of the Internet and the resulting overexposure to rapidly disseminating false information is a problem.

The third major finding of this study is that misinformation regarding COVID-19 transmission is significantly and positively correlated with life satisfaction. This finding demonstrates that individuals with a high level of misinformation report greater levels of life satisfaction. This is a surprising discovery. Another finding of ours is that ignorance about COVID-19 transmission misinformation predicts adult life satisfaction. The quality of life of a person who is aware of COVID-19 transmission misinformation is enhanced. Maintaining a consistent flow of information regarding COVID-19 can be extremely stressful and frustrating, resulting in information overload and making it difficult to determine whether the information is credible and useful (Mohammed et al., 2021). Individuals will experience greater life satisfaction if they can distinguish between correct and incorrect knowledge. Adults’ interests and behaviors are consistent with COVID-19 disinformation, ensuring their happiness in life. Incorporating pepper into meals, for instance, may aid in preventing COVID-19 infection, whereas consuming garlic may aid in preventing COVID-19 contraction. There is a positive correlation between COVID-19 transmitted misinformation and life satisfaction because individuals with high exposure to COVID-19 transmitted misinformation (e.g., prevention, disease transmission methods) equip themselves and take precautions to be prepared for the worst-case scenario, thus increasing their level of life satisfaction.

False information regarding COVID-19 transmission is another source of evidence for a connection between COVID-19 stress and life satisfaction. Misinformation regarding COVID-19 transmission mediated the association between COVID-19 stress and life satisfaction. The partial mediation suggests that misinformation about COVID-19 transmission indirectly affected life satisfaction. According to Lockyer et al. (2021), when participants were asked about their associations with transmission misinformation, they pondered whether they disliked or appreciated it. As a result of ignoring the stories, the participants were unsure of whom to believe or listen to, indicating that they were unable to disprove them. According to Bermes (2021), individuals experiencing emotional stress are more likely to spread false information. This is consistent with our study’s findings. In addition, we discovered a correlation between COVID-19 transmission misinformation and life satisfaction. This implies that individuals can increase their level of life satisfaction if they spread misinformation in a positive manner and misinterpret information as expected. Indicating that COVID-19 transmission misinformation moderates the effect of COVID-19 stress on life satisfaction, our findings contribute to the body of knowledge concerning life satisfaction. Previous models did not take into account the role of COVID-19 transmission misinformation as a mediator in the relationship between COVID-19 stress and life satisfaction; this knowledge will facilitate the development of more targeted therapies.

### Implication

Despite a number of restrictions, the research results contribute to the development and development of future theories and methods addressing mental health concerns in the Vietnamese community. The discoveries are also both theoretical and practical. Our data give considerable evidence of interactions between COVID-19 transmission misinformation, stress, and Vietnamese citizens’ satisfaction with life. Our study examined the impact of COVID-19 transmission misinformation as a mediating factor in the link between stress and satisfaction with life.

The findings have several practical ramifications and contribute to a comprehensive knowledge of the detrimental effect of COVID-19 transmission disinformation on stress. During the stressful epidemic period, people face many challenges and the level of human stress during the pandemic increases (Cao et al., 2020; Wang et al., 2021). Previous research has also shown that emergencies such as pandemics have many problems that significantly affect the emotional state of people causing stress, perceived stress has a negative relationship with the health of people and satisfaction with life (Gori et al., 2020). In this regard, many measures have been proposed to support mental health care during the pandemic (Madsen et al., 2020; Ogueji et al., 2021).^,^ A stress management strategy that focuses on stress reevaluation is a promising way or possible way to view stress-is-increasing the mindset of viewing stress as an enhanced impact on functioning, performance, and health healthy (Hagger et al., 2020). Moreover, COVID-19 transmission misinformation increased stress levels. To avoid misleading and ambiguous language, and to prevent any unclear content from becoming disinformation, it is vital to stress each element in a piece of health information (Chen et al., 2021). Chen et al. (2021) suggested that one strategy to avoid misinformation is to provide timely, targeted, and accurate information based on forecasts of the potential COVID-19 epidemic situation. According to Patwary et al. (2021), if social media businesses filter content and eliminate disinformation, these activities could help alleviate global worry and stress.

Additionally, satisfaction with life is evaluated by an individual’s assessment of the difference between what they have and what they expect. In other words, it is a subjective assessment of the extent to which an individual’s needs, aspirations, and desires are met. Individuals who retain a feeling of balance in their life and make favorable assessments of their standards and aspirations are considered to be extremely content. As a result, sources that clearly identify the veracity of information are essential to ensure that individuals understand what information is accurate and what is not, therefore maximizing satisfaction with life.

### Limitations and future directions

This is the first study to show a link between COVID-19 stress and COVID-19 transmission disinformation and life happiness, indicating several new research avenues and areas of interest for future research. There are several drawbacks to the study. First, we recruit participants *via* convenience sampling, limiting the study’s applicability to a representative Vietnamese community. Future research should study if the current model applies to individuals of all ages, as this will enable researchers to better comprehend the relationship between COVID-19 Stress and Satisfaction with Life. Second, the COVID-19 Transmission Misinformation scale by Bok et al. (2021) contains an item on 5G networks; however, 5G networks are uncommon in Vietnam, and the research team has not adjusted the content of the item to suit the development in Vietnam. Future studies should consider adjusting the content of items to suit the context at the time of research. Third, the data was collected during the time people are restricted from leaving their homes because of COVID-19, we conduct the survey online and where we distribute the ballots there are more females than men, which may affect the research results. Future studies may retest with an even sex distribution. In addition, the age of the participants is unevenly spread because the number of social network users is small, with the bulk of users being between 19 and 44 years old. On the other side, the research team has included a vast group of ages in the demographic, resulting in certain age groups with no respondents in the survey sample. Fourth, the present research has not examined other characteristics that may predict COVID-19 Stress among Vietnamese adults (e.g., education, living status, and socioeconomic position); nevertheless, these aspects may be addressed in the future through research efforts.

## Conclusion

The numbers indicate that the COVID-19 pandemic has increased the stress levels of the general populace when prior study findings and our new findings are combined. To reduce negative effects on adult life satisfaction, it is essential to analyze the impact of disinformation on prominent social media sites accessible to adults in order to produce timely and concise reports. The results imply a connection between COVID-19 stress, life satisfaction, and COVID-19 transmission misinformation. Recent research indicates that transmission misinformation about COVID-19 transmission acts as a mediator between COVID-19 stress and life satisfaction, implying that an increase in transmission misinformation correlates with higher COVID-19 stress and higher Satisfaction with Life. The data indicate how concerned people are about the effects of COVID-19 transmission misinformation on their satisfaction, suggesting that adults become more stressed when exposed to COVID-19 transmission misinformation. The findings contribute to an important body of research and contribute to a better understanding of the positive effect of misinformation on life satisfaction. This is the first study in Vietnam to examine the relationship between COVID-19-related stress, misinformation about COVID-19 transmission, and life satisfaction. This will be one of the sources of information for the future comparative study based on our findings, regardless of any discrepancies in findings.

## Data availability statement

The original contributions presented in the study are included in the article/supplementary material, further inquiries can be directed to the corresponding author.

## Ethics statement

The current study complied with the Declaration of Helsinki’s standards for human subjects’ research. The Ethics Committee of the Department of Science and Technology – the Ho Chi Minh City University of Education (under the Vietnamese Ministry of Education) accepted the research by granting it a confirmation code NV2021.19.03. DH on September 1, 2021. The patients/participants provided their written informed consent to participate in this study.

## Author contributions

PN, SH, and V-LT-C contributed to the conception and design of the study. V-LT-C, GN, NN, and PL organized the database. V-LT-C and TL performed the statistical analysis. TL, NN, and PL wrote the first draft of the manuscript. All authors contributed to manuscript revision, read, and approved the submitted version.

## Conflict of interest

The authors declare that the research was conducted in the absence of any commercial or financial relationships that could be construed as a potential conflict of interest.

## Publisher’s note

All claims expressed in this article are solely those of the authors and do not necessarily represent those of their affiliated organizations, or those of the publisher, the editors and the reviewers. Any product that may be evaluated in this article, or claim that may be made by its manufacturer, is not guaranteed or endorsed by the publisher.

## References

[ref1] Abdallah T. (1998). The satisfaction with life scale (SWLS): psychometric properties in an Arabic-speaking sample. Int. J. Adolesc. Youth 7, 113–119. doi: 10.1080/02673843.1998.9747816

[ref2] Ahmed W. Vidal-Alaball J. Downing J. López Seguí F. (2020). COVID-19 and the 5G conspiracy theory: social network analysis of twitter data. J. Med. Internet Res. 22, e19458–e19459. doi: 10.2196/19458, PMID: 32352383 PMC7205032

[ref3] Akinwande M. O. Dikko H. G. Samson A. (2015). Variance inflation factor: as a condition for the inclusion of suppressor variable (s) in regression analysis. Open J. Stat. 05, 754–767. doi: 10.4236/ojs.2015.57075

[ref4] Ali S. (2022). Combatting against COVID-19 & misinformation: a systematic review. Hu Arenas 5, 337–352. doi: 10.1007/s42087-020-00139-1

[ref5] Allison PD. (1999). Multiple Regression: A Primer. Thousand Oaks, CA: Pine Forge Press.

[ref6] Anand K. B. Karade S. Sen S. Gupta R. M. (2020). SARS-CoV-2: camazotz's curse. Med. J. Arm. For. India 76, 136–141. doi: 10.1016/j.mjafi.2020.04.008, PMID: 32341622 PMC7183968

[ref7] Anastasiades E. Argyrides M. Mousoulidou M. (2021). Misinformation about COVID-19: psychological insights. Encyclopedia 1, 1200–1214. doi: 10.3390/encyclopedia1040091

[ref8] Asmundson G. Paluszek M. Landry C. Rachor G. McKay D. Taylor S. (2020). Do pre-existing anxiety-related and mood disorders differentially impact COVID-19 stress responses and coping? J. Anxiety Disord. 74, 102271–102276. doi: 10.1016/j.janxdis.2020.102271, PMID: 32673930 PMC7342169

[ref9] Barua Z. Barua S. Aktar S. Kabir N. Li M. (2020). Effects of misinformation on COVID-19 individual responses and recommendations for resilience of disastrous consequences of misinformation. Prog. Dis. Sci. 8:100119. doi: 10.1016/j.pdisas.2020.100119, PMID: 34173443 PMC7373041

[ref10] Basch C. Hillyer G. Meleo-Erwin Z. Jaime C. Mohlman J. Basch C. (2020). Preventive behaviors conveyed on YouTube to mitigate transmission of COVID-19: cross-sectional study. JMIR Public Health Surveill. 6, e18807–e18806. doi: 10.2196/18807, PMID: 32240096 PMC7124952

[ref11] Bermes A. (2021). Information overload and fake news sharing: a transactional stress perspective exploring the mitigating role of consumers’ resilience during COVID-19. J. Retail. Consum. Serv. 61, 102555–102510. doi: 10.1016/j.jretconser.2021.102555

[ref12] Bok S. Martin D. E. Acosta E. Lee M. Shum J. (2021). Validation of the COVID-19 transmission misinformation scale and conditional indirect negative effects on wearing a mask in public. Int. J. Environ. Res. Public Health 18, 1–23. doi: 10.3390/ijerph182111319, PMID: 34769835 PMC8583109

[ref13] Bukhari S. R. Asim S. Ghani M. U. Muhammad S. Gani N. Ashraf W. (2021). The effect of perceived stress on satisfaction with life of general population in the time of COVID-19 pandemic. Rawal Med. J. 46, 11–13. https://www.bibliomed.org/mnsfulltext/27/27-1604389251.pdf?1648042261

[ref14] Cao W. Fang Z. Hou G. Han M. Xu X. Dong J.. (2020). The psychological impact of the COVID-19 epidemic on college students in China. Psychiatry Res. 287, 112934–112935. doi: 10.1016/j.psychres.2020.112934, PMID: 32229390 PMC7102633

[ref15] Chen K. Luo Y. Hu A. Zhao J. Zhang L. (2021). Characteristics of misinformation spreading on social media during the COVID-19 outbreak in China: a descriptive analysis. Risk Manag. Health. Pol. 14, 1869–1879. doi: 10.2147/RMHP.S312327, PMID: 34007225 PMC8121282

[ref16] Cuan-Baltazar J. Muñoz-Perez M. Robledo-Vega C. Pérez-Zepeda M. Soto-Vega E. (2020). Misinformation of COVID-19 on the internet: infodemiology study. JMIR Public Health Surveill. 6, e18444–e18446. doi: 10.2196/18444, PMID: 32250960 PMC7147328

[ref17] Demir Y. Pinar O. R. U. Ç. Kilinç Z. A. Özpinar S. (2021). The perceived stress and its effects on satisfaction with life: emergency department staff during COVID-19 pandemic. Fenerbahçe Üniversitesi Sağlık Bilimleri Dergisi 1, 256–268. https://dergipark.org.tr/en/download/article-file/2070528

[ref18] Diener E. Emmons R. A. Larsen R. J. Griffin S. (1985). The satisfaction with life scale. J. Pers. Assess. 49, 71–75. doi: 10.1207/s15327752jpa4901_13 16367493

[ref19] Duong C. D. (2021). The impact of fear and anxiety of COVID-19 on life satisfaction: psychological distress and sleep disturbance as mediators. Pers. Individ. Differ. 178:110869. doi: 10.1016/j.paid.2021.110869 PMC9665951 36406744

[ref20] Dymecka J. Gerymski R. Machnik-Czerwik A. Derbis R. Bidzan M. (2021). Fear of COVID-19 and life satisfaction: the role of the health-related hardiness and sense of coherence. Front. Psychol. 12, 1–9. doi: 10.3389/fpsyt.2021.712103, PMID: 34790135 PMC8591072

[ref21] Elbarazi I. Saddik B. Grivna M. Aziz F. Elsori D. Stip E.. (2022). The impact of the COVID-19 “Infodemic” on well-being: a cross-sectional study. J. Multidiscip. Healthc. 15, 289–307. doi: 10.2147/JMDH.S346930, PMID: 35228802 PMC8881924

[ref22] Field A. (2016). An Adventure in Statistics: The Reality Enigma. Los Angeles, CA: Sage.

[ref23] Garcini L. M. Rosenfeld J. Kneese G. Bondurant R. G. Kanzler K. E. (2021). Dealing with distress from the COVID-19 pandemic: mental health stressors and coping strategies in vulnerable latinx communities. Health Soc. Care Community 30, 284–294. doi: 10.1111/hsc.13402, PMID: 33894080 PMC8251305

[ref24] Gori A. Topino E. Di Fabio A. (2020). The protective role of life satisfaction, coping strategies and defense mechanisms on perceived stress due to COVID-19 emergency: a chained mediation model. PLoS One 15, e0242402–e0242411. doi: 10.1371/journal.pone.0242402, PMID: 33186367 PMC7665746

[ref25] Groarke J. M. Berry E. Graham-Wisener L. McKenna-Plumley P. E. McGlinchey E. Armour C. (2020). Loneliness in the UK during the COVID-19 pandemic: cross-sectional results from the COVID-19 psychological wellbeing study. PLoS One 15, 1–27. doi: 10.31234/osf.io/j2pce PMC7513993 32970764

[ref26] Hagger M. S. Keech J. J. Hamilton K. (2020). Managing stress during the coronavirus disease 2019 pandemic and beyond: reappraisal and mindset approaches. Stress. Health 36, 396–401. doi: 10.1002/smi.2969, PMID: 32588961 PMC7361383

[ref27] Hair J Guyot D Westad F. (2010). Multivariate Data Analysis. Upper Saddle River, NJ: Pearson Prentice Hall.

[ref28] Hayes A. F. (2017). *Introduction to Mediation*, *Moderation*, *and Conditional Process Analysis: A Regression-Based Approach*. New York City, NY: Guilford Publications.

[ref29] Jacobson N. C. Lekkas D. Price G. Heinz M. V. Song M. O’Malley A. J.. (2020). Flattening the mental health curve: COVID-19 stay-at-home orders are associated with alterations in mental health search behavior in the United States. JMIR Ment. Health 7, 1–44. doi: 10.2196/19347, PMID: 32459186 PMC7265799

[ref30] Jain P. (2021). The COVID-19 pandemic and positive psychology: the role of news and trust in news on mental health and well-being. J. Health Commun. 26, 317–327. doi: 10.1080/10810730.2021.1946219, PMID: 34185615

[ref31] Joshanloo M. Jovanović V. (2020). The relationship between gender and life satisfaction: analysis across demographic groups and global regions. Arch. Females Ment. Health 23, 331–338. doi: 10.1007/s00737-019-00998-w, PMID: 31482245

[ref32] Kalidoss N. Banerjee D. (2022). Misinformation, mental well-being and third wave. News India Exp. https://www.newindianexpress.com/opinions/2022/jan/15/misinformation-mental-well-being-and-third-wave-2406951.html

[ref33] Karatzias T. Shevlin M. Murphy J. McBride O. Ben-Ezra M. Bentall R. P.. (2020). Posttraumatic stress symptoms and associated comorbidity during the COVID-19 pandemic in Ireland: a population-based study. J. Trauma. Stress. 33, 365–370. doi: 10.1002/jts.22565, PMID: 32662129 PMC7405473

[ref34] Kecojevic A. Basch C. Sullivan M. Davi N. (2020). The impact of the COVID-19 epidemic on mental health of undergraduate students in New Jersey, cross-sectional study. PLoS One 15, e0239696–e0239616. doi: 10.1371/journal.pone.0239696, PMID: 32997683 PMC7526896

[ref35] Köker S. (1991). Comparison of Satisfaction With Life Levels of Normal and Problematic Adolescents. Unpublished Master's Thesis, Ankara University Institute of Social Sciences, Ankara.

[ref36] Kruger P. S. Engelbrecht S. W. (2010). Happiness around the world the paradox of happy peasants and miserable millionaires. Appl. Res. Qual. Life 5, 165–169. doi: 10.1007/s11482-010-9100-z

[ref37] Laato S. Islam A. N. Islam M. N. Whelan E. (2020). What drives unverified information sharing and cyberchondria during the COVID-19 pandemic? Eur. J. Inf. Syst. 29, 288–305. doi: 10.1080/0960085X.2020.17706

[ref38] Lee C. W. Lin L. C. Hung H. C. (2021). Art and cultural participation and satisfaction with life in adults: the role of physical health, mental health, and interpersonal relationships. Front. Public Health 8, 1–9. doi: 10.3389/fpubh.2020.582342, PMID: 33558844 PMC7864897

[ref39] Lockyer B. Islam S. Rahman A. Dickerson J. Pickett K. Sheldon T.. (2021). Understanding COVID-19 misinformation and vaccine hesitancy in context: findings from a qualitative study involving citizens in Bradford. UK Health Exp. 24, 1158–1167. doi: 10.1111/hex.13240, PMID: 33942948 PMC8239544

[ref40] Luqman A. Cao X. Ali A. Masood A. Yu L. (2017). Empirical investigation of Facebook discontinues usage intentions based on SOR paradigm. Comput. Hum. Behav. 70, 544–555. doi: 10.1016/j.chb.2017.01.020

[ref41] Madsen M. M. Dines D. Hieronymus F. (2020). Optimizing psychiatric care during the COVID-19 pandemic. Acta Psychiatr. Scand. 142, 70–71. doi: 10.1111/acps.13176, PMID: 32323297 PMC7264740

[ref42] Mohammed M. Sha’aban A. Jatau A. I. Yunusa I. Isa A. M. Wada A. S.. (2021). Assessment of COVID-19 information overload among the general public. J. Rac. Ethn. Health Disp. 9, 184–192. doi: 10.1007/s40615-020-00942-0, PMID: 33469869 PMC7815186

[ref43] National Literacy Trust (2018). Fake news and critical literacy: the final report of the commission on fake news and the teaching of critical literacy in schools. Nat. Lit. Trust. https://literacytrust.org.uk/research-services/research-reports/fake-news-and-critical-literacy-final-report/

[ref44] Nettleton D. (2014). “Chapter 6 – selection of variables and factor derivation in “commercial data mining” processing,” in Commercial Data Mining Processing, Analysis and Modeling for Predictive Analytics Projects: The Savvy Manager’s Guides. eds. D. Nettleton (Burlington, MA: Morgan Kaufmann Publishers Elsevier Inc.), 79–104. doi: 10.1016/B978-0-12-416602-8.00006-6

[ref45] Nguyen T. M. Le G. N. H. (2021). The influence of COVID-19 stress on psychological well-being among Vietnamese adults: the role of self-compassion and gratitude. Traumatology 27, 86–97. doi: 10.1037/trm0000295

[ref46] Ocaña-Moral M. T. Gavín-Chocano Ó. Pérez-Navío E. Martínez-Serrano M. D. C. (2021). Relationship among perceived stress, life satisfaction and academic performance of education sciences students of the University of Jaén after the COVID-19 pandemic. Educ. Sci. 11, 1–15. doi: 10.3390/educsci11120802

[ref47] Ogueji I. A. Okoloba M. M. Demoko Ceccaldi B. M. (2021). Coping strategies of individuals in the United Kingdom during the COVID-19 pandemic. Curr. Psychol. 1-7, 1–7. doi: 10.1007/s12144-020-01318-7, PMID: 33424202 PMC7779093

[ref48] Oh J. Neal Z. P. (2021). Two’s company, but Four’sa crowd: the relationship among COVID-19 stress, household size, and life satisfaction. Collab. Psychol. 7, 1–15. doi: 10.1525/collabra.24923

[ref49] Otu A. Charles C. H. Yaya S. (2020). Mental health and psychosocial well-being during the COVID-19 pandemic: the invisible elephant in the room. Int. J. Ment. Heal. Syst. 14, 1–5. doi: 10.1186/s13033-020-00371-w, PMID: 32514302 PMC7257210

[ref50] Patwary M. M. Bardhan M. Browning M. H. Disha A. S. Haque M. Z. Billah S. M.. (2021). Association between perceived trusted of COVID-19 information sources and mental health during the early stage of the pandemic in Bangladesh. Healthcare 10, 1–17. doi: 10.3390/healthcare10010024, PMID: 35052191 PMC8775621

[ref51] Pfefferbaum B. North C. S. (2020). Mental health and the Covid-19 pandemic. N. Engl. J. Med. 383, 510–512. doi: 10.1111/inm.12726 32283003

[ref52] Preetz R. Filser A. Brömmelhaus A. Baalmann T. Feldhaus M. (2021). Longitudinal changes in life satisfaction and mental health in emerging adulthood during the COVID-19 pandemic. Risk Prot. Fact. Emerg. Adulth. 9, 602–617. doi: 10.1177/21676968211042109

[ref53] Priego-Parra B. A. Triana-Romero A. Pinto-Gálvez S. M. Ramos C. D. Salas-Nolasco O. Reyes M. M.. (2020). Anxiety, depression, attitudes, and internet addiction during the initial phase of the 2019 coronavirus disease (COVID-19) epidemic: a cross-sectional study in Mexico. MedRxiv 602–617. doi: 10.1101/2020.05.10.20095844

[ref54] Quan N. K. Le T. N. Khanh P. N. Q. Huy N. T. (2021a). COVID-19 timeline of Vietnam: important milestones through four waves of the pandemic and lesson learned. Front. Public Health 9, 1–27. doi: 10.3389/fpubh.2021.709067, PMID: 34900885 PMC8651614

[ref55] Quan T. A. Quynh N. N. Huyen N. T. N. (2021b). Sức khoẻ tinh thần tại Việt Nam trong thời kì COVID-19 [Mental health in Vietnam during COVID-19]. OSF. doi: 10.31219/osf.io/3unvp

[ref56] Raza S. H. Haq W. Sajjad M. (2020). COVID-19: a psychosocial perspective. Front in Psychology 11, 1–12. doi: 10.3389/fpsyg.2020.554624, PMID: 33335494 PMC7736613

[ref57] Rosenberg H. Syed S. Rezaie S. (2020). The twitter pandemic: the critical role of twitter in the dissemination of medical information and misinformation during the COVID-19 pandemic. Can. J. Emerg. Med. 22, 418–421. doi: 10.1017/cem.2020.361, PMID: 32248871 PMC7170811

[ref58] Shrout P. E. Bolger N. (2002). Mediation in experimental and nonexperimental studies: new procedures and recommendations. Psychol. Methods 7, 422–445. doi: 10.1037/1082-989X.7.4.422, PMID: 12530702

[ref59] Şimşek E. (2011). *The Effects of Organizational Communication and Personality Traits on Satisfaction With Life*. *Unpublished Doctoral Thesis*. Eskisehir/Turkey: Anadolu Universities.

[ref60] Su Z. McDonnell D. Wen J. Kozak M. Abbas J. Šegalo S.. (2021). Mental health consequences of COVID-19 media coverage: the need for effective crisis communication practices. Glob. Health 17, 1–8. doi: 10.1186/s12992-020-00654-4, PMID: 33402169 PMC7784222

[ref61] Taylor S. Landry C. A. Paluszek M. M. Fergus T. A. McKay D. Asmundson G. J. (2020). COVID stress syndrome: concept, structure, and correlates. Depress. Anxiety 37, 706–714. doi: 10.1002/da.23071, PMID: 32627255 PMC7362150

[ref62] Taylor S. Landry C. Paluszek M. Fergus T. McKay D. Asmundson G. (2020). Development and initial validation of the COVID stress scales. J. Anxiety Disord. 72, 102232–102237. doi: 10.1016/j.janxdis.2020.102232, PMID: 32408047 PMC7198206

[ref63] Than H. M. Nong V. M. Nguyen C. T. Dong K. P. Ngo H. T. Doan T. T.. (2020). Mental health and health-related quality-of-life outcomes among frontline health workers during the peak of COVID-19 outbreak in Vietnam: a cross-sectional study. Risk Manag. Healthc. Policy 13, 2927–2936. doi: 10.2147/RMHP.S280749, PMID: 33324126 PMC7733435

[ref64] Tharp D. T. Parks-Stamm E. J. Kitces M. Lurtz M. (2021). Gender differences in COVID-19-related stress and relationships with life satisfaction among financial advisors. Fin. Plan. Rev. 4, 1–14. doi: 10.1002/cfp2.1129

[ref65] Torales J. O’Higgins M. Castaldelli-Maia J. M. Ventriglio A. (2020). The outbreak of COVID-19 coronavirus and its impact on global mental health. Int. J. Soc. Psychiatry 66, 317–320. doi: 10.1177/0020764020915212 32233719

[ref66] Tran B. X. Nguyen H. T. Le H. T. Latkin C. A. Pham H. Q. Vu L. G.. (2020). Impact of COVID-19 on economic well-being and quality of life of the Vietnamese during the national social distancing. Risk Manag. Healthc. Policy 13, 2927–2936. doi: 10.2147/RMHP.S280749, PMID: 33324126 PMC7733435

[ref67] Umucu E. Tansey T. N. Brooks J. Lee B. (2021). The protective role of character strengths in COVID-19 stress and well-being in individuals with chronic conditions and disabilities: an exploratory study. Rehab. Couns. Bull. 64, 67–74. doi: 10.1177/0034355220967093

[ref68] van der Laan S. E. Finkenauer C. Lenters V. C. Van Harmelen A. L. van der Ent C. K. Nijhof S. L. (2021). Gender-specific changes in life satisfaction after the COVID-19–related lockdown in Dutch adolescents: a longitudinal study. J. Adolesc. Health 69, 737–745. doi: 10.1016/j.jadohealth.2021.07.013, PMID: 34446346 PMC8460170

[ref69] Wang Y. Di Y. Ye J. Wei W. (2021). Study on the public psychological states and its related factors during the outbreak of coronavirus disease 2019 (COVID-19) in some regions of China. Psychol. Health Med. 26, 13–22. doi: 10.1080/13548506.2020.1746817, PMID: 32223317

[ref70] World Health Organization (2020). Coronavirus Disease 2019 (COVID-19) World Health Organization *Situation Report 86*: https://apps.who.int/iris/handle/10665/331784.

[ref71] World Health Organization (2022). COVID-19 in Viet Nam World Health Organization Situation Report 81: https://www.who.int/vietnam/internal-publications-detail/covid-19-in-viet-nam-situation-report-81.

[ref72] Xiong J. Lipsitz O. Nasri F. Lui L. M. Gill H. Phan L.. (2020). Impact of COVID-19 pandemic on mental health in the general population: a systematic review. J. Affect. Disord. 277, 55–64. doi: 10.1016/j.jad.2020.08.001, PMID: 32799105 PMC7413844

[ref73] Yalçın İ. Can N. Mançe Çalışır Ö. Yalçın S. Çolak B. (2022). Latent profile analysis of COVID-19 fear, depression, anxiety, stress, mindfulness, and resilience. Curr. Psychol. 41, 459–469. doi: 10.1007/s12144-021-01667-x, PMID: 33821112 PMC8012016

[ref74] Zandifar A. Badrfam R. (2020). Iranian mental health during the COVID-19 epidemic. Asian J. Psychiatr. 51, 101990–101992. doi: 10.1016/j.ajp.2020.101990, PMID: 32163908 PMC7128485

[ref75] Zarocostas J. (2020). How to fight an infodemic. Lancet 395:676. doi: 10.1016/S0140-6736(20)30461-X, PMID: 32113495 PMC7133615

